# Origin-dependence of variation in seed morphology, mineral composition and germination percentage in *Gynandropsis gynandra* (L.) Briq. accessions from Africa and Asia

**DOI:** 10.1186/s12870-020-02364-w

**Published:** 2020-04-15

**Authors:** Jelila S. Blalogoe, Alfred O. Odindo, E. O. Dêêdi Sogbohossou, Julia Sibiya, Enoch G. Achigan-Dako

**Affiliations:** 1 Laboratory of Genetics, Horticulture and Seed Science, Faculty of Agronomic Sciences, University of Abomey-Calavi, BP 526 Abomey-Calavi, Republic of Benin; 2 Discipline of Plant Breeding, School of Agricultural, Earth and Environmental Sciences, University of KwaZulu-Natal, Private Bag X01, Scottsville 3209, Pietermaritzburg, Republic of South Africa; 3 Crop Science, School of Agricultural, Earth and Environmental Sciences, University of KwaZulu-Natal, Pietermaritzburg, Republic of South Africa; 4 Biosystematics Group, Wageningen University, Postbus 647 6700AP, Wageningen, The Netherlands

**Keywords:** Characterization, *Cleome gynandra*, Germination, Mineral element contents, Seed morphology, Seed viability, Scanning Electron microscope

## Abstract

**Background:**

Spider plant [*Gynandropsis gynandra* (L.) Briq.], an economically promising African leafy vegetable, characterized for leaf yield components and nutritive quality, exhibits poor seed germination that hinders a wider expansion of the crop in urban and periurban horticultural systems. So far, there is little information pertaining to seed morphological traits and mineral elements content that may be associated with higher seed germination. This research investigated the hypothesis that spider plants from different geographical areas exhibited differences in seed mineral composition, morphological traits, and germination capacity. To this end, twenty-nine accessions of *Gynandropsis gynandra* from West and East-Southern Africa, and Asia were screened for variation in seed size (area, perimeter, length, width), 10-seed weight, mean germination time, germination percentage and mineral content variations. The scanning electron microscopy (SEM), light microscopy and energy dispersive spectroscopy (EDS) solution were used to study seed morphology and mineral composition.

**Results:**

We show for the first time the external and internal structure of the seeds of *Gynandropsis gynandra* and measured eight mineral elements, including carbon (C), oxygen (O), magnesium (Mg), aluminium (Al), phosphorus (P), sulphur (S), potassium (K) and calcium (Ca). The accessions differed significantly (*p* < 0.001) with respect to seed size (area, perimeter, length, width), 10-seed weight, mean germination time and germination percentage. The hierarchical cluster analysis based on fourteen variables grouped the accessions into three distinct clusters, partially dependent on their geographical origin. Asian accessions exhibited smaller seeds and recorded higher values in terms of germination percentage. West African accessions had bigger seeds but with lower germination percentage. Variation in minerals such as potassium, carbon, and calcium content showed different patterns according to geographical origins.

**Conclusion:**

Smaller seeds in *G. gynandra* exhibited better germination capacity. The Asian germplasm is a potential source of cultivars with a higher germination percentage for improving seed quality in the species.

## Background

Successful breeding programmes and crop production require good quality seeds for increased yield to ensure food and nutritional security for the growing populations [1, 2]. Seed germination and seedling emergence are critical phases in the development of plants and this implies that seed vigour is an important trait for the selection of important crop cultivars [3, 4]. The capacity of a seed to germinate quickly and competitively depends on the genetic and physiological constitution of the seed [5, 6]. In addition, seed size at maturity is an important physical indicator of seed quality which can affect vegetative growth and is frequently related to yield [1]. It is commonly known that because of the larger store of carbohydrate in the seed endosperm or cotyledons, seedlings from larger seeds have a better start in life and better field performance than smaller seeds [7]. Morphological measurements including length, width, area, perimeter and weight are important parameters for determining the size and shape of seeds [8]. Seed shape and size can influence water imbibition, seed moisture content and consequently seed germination and quality [9–11]. For instance, Gholami and al [12] observed an increase in germination as well as a greater speed of germination in larger seeds compared with small seeds in the common bean (*Phaseolus vulgaris* L.). It was shown that larger seeds of *Amaranthus* spp. possessed higher physiological quality [13] than smaller seeds.

In addition, seed mineral composition also determines plant establishment and growth. Seeds contain several macronutrients like phosphorus (P) and micronutrients, including Zinc (Zn), Boron (B), Molybdenum (Mo), Selenium (Se), copper (Cu), cobalt (Co) which are important for seed germination, seedling emergence and seed vigour [3, 14, 15]. For instance, it was observed that annual pasture legume yields are positively correlated with phosphorus concentration in seeds [16].

Breeding for grain yield, seed size, seed mineral content and seed vigour requires a fundamental assessment of the seed metrics, mineral composition, and germination capacity. In *Gynandropsis gynandra* (Cleomaceae), low seed germination was reported by growers as a major constraint limiting the species’ productivity [17]. Although *Gynandropsis gynandra* is a leafy vegetable with high nutritional values [18–20], the leaf yield is not only dependent on the leaf components but also on the seed quality and mineral composition. Characterization studies [21–23] focused on traits including leaf number, number of branches, stem colour and leaf area. Variation in seed morphology has been rarely documented although a large genetic diversity among accessions were frequently reported for morphological traits [19, 20, 23, 24], leaf mineral composition [19] and secondary metabolites [20]. However, variation in the quality and mineral composition of the seeds of *G. gynandra* have not yet been documented. Information on the variability in morphological traits and mineral composition among accessions from different regions could be useful to improve seed quality and develop a strong breeding agenda.

The objectives of this study are therefore (i) to screen seeds of *G. gynandra* from different geographical regions for their mineral composition and (ii) to assess seed phenotypic diversity and variation in mineral content among accessions and in relation to seed germination. The study hypothesized that mineral composition and seed size in *Gynandropsis gynandra* vary among accessions from different geographical regions, and large seeds with high phosphorus content germinate better than small seeds with low phosphorus content.

## Results

### External and internal structures of *Gynandropsis gynandra* seeds

**Fig. 1:**
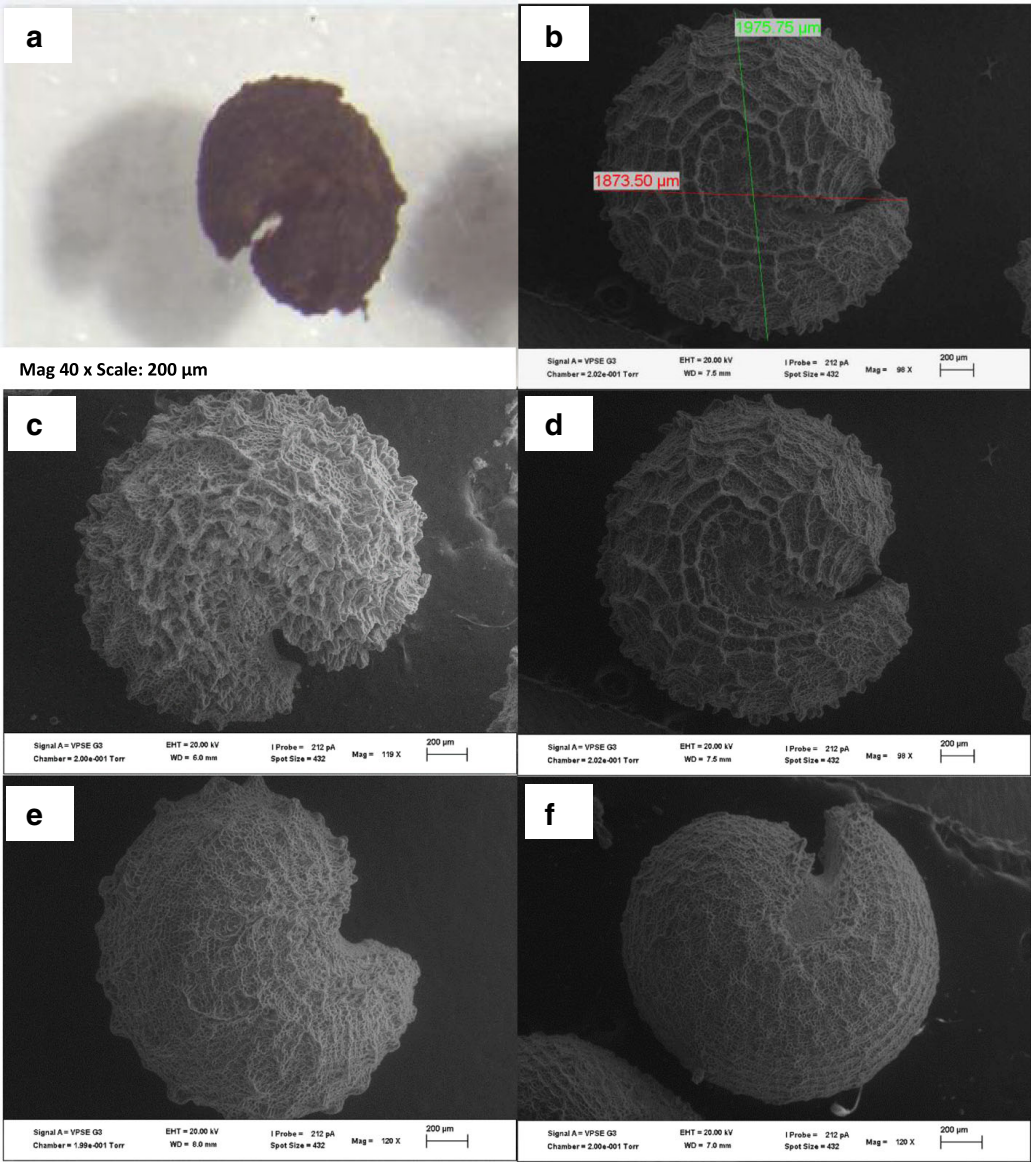
SEM of *Gynandropsis gynandra* accessions. **a:** Light microscopy showing seed colour;** b**: Seed image analysis (in green the seed length and in red the seed width); **c**: Fairly round shape with very rough seed surface; **d**: Round shape with very rough seed surface; **e**: Fairly round shape with slightly rough seed surface **f**: Round shape with slightly rough seed surface

**Fig. 2:**
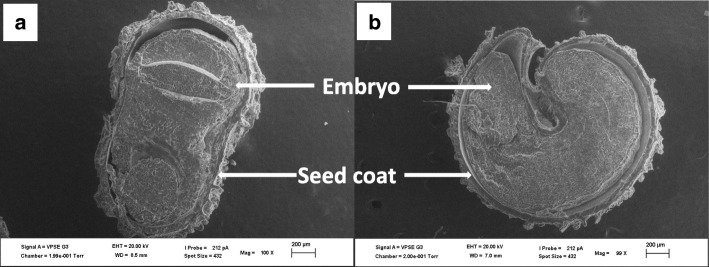
*Gynandropsis gynandra* internal seed morphology under Scanning Electron Microscopy. **a**: Seed cross section **b**: Seed longitudinal section

**Fig. 3:**
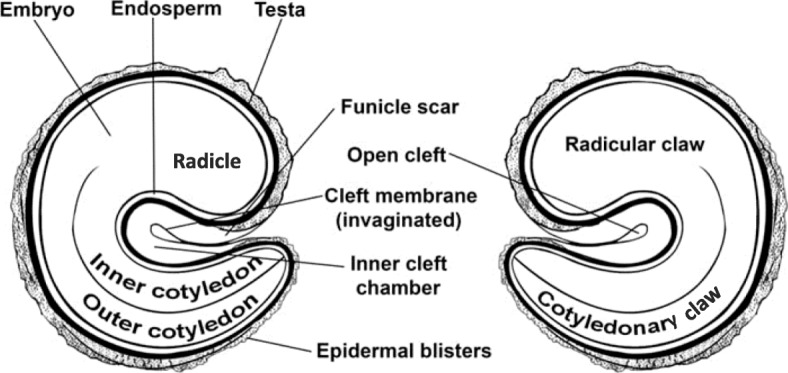
Illustration of *Gynandropsis gynandra* seed in longitudinal section as viewed in light microscope: adapted from Iltis et al. [25]

*Gynandropsis gynandra* seeds are generally brown or black in colour (Fig. 1 a and b). The seeds are round or fairly round depending on the accession and pointed at the apical region where the radicle is located. The hilum is located at the center of the seed. The surface of the seed is rough with small rounded or ocellated depressions and ridges on the whole surface of the seed. Spider plant accessions split into two major seed types (Table 1) based on the seed surface. The first one consisted of thirteen accessions with slightly rough seed surface (Fig. 1 c and d) and the second consisted of sixteen accessions with very rough seed surface (Fig. 1 e and f). Illustrations of cross and longitudinal sections, based on observations from the Scanning Electron Microscopy (SEM) showed the embryo and the seed coat (Fig. 2) but not the overall organization of the seed. The longitudinal section of seeds observed under a light microscope revealed the overall organization of the spider plant seeds (Fig. 3). The embryo consisted of a hypocotyl-radicle axis and two cotyledons and the endosperm in the micropylar region.

**Table 1:** Distribution of *Gynandropsis gynandra* accessions based of seed surface roughness

Seed surface roughness	Accessions name
Slightly rough seed surface	ODS-15-020, ODS-15-061, ODS-15-100, HBY 2307/b, KSI, TOT7196, TOT6439, ODS-15-121, TOT7200SC, TOT8931, TOT1048, ELG1907A, BAR1807B
Very rough seed surface	ODS-15-115, TOT4976, TOT7486, ODS-15-019, TOT7505, KF 07, TOT5799, ODS-15-013, TOT8886, TOT7198, ODS-15-045, TOT3536

### Variation in seeds quantitative morphological traits

**Fig. 4:**
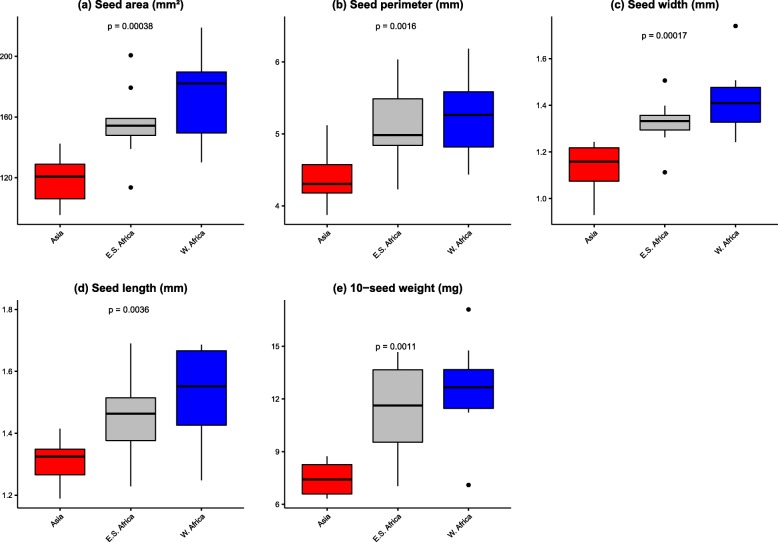
Boxplot showing the variation in seeds morphological descriptors among Asian (*n = 10*), East–Southern African (*n* = 9) and West African (*n* = 10) accessions of *Gynandropsis gynandra*

The analysis of variance (ANOVA) showed highly significant differences among the spider plant accessions for morphological traits such as seed area, seed perimeter, seed length, seed width, and 10-seed weight (Supplementary file 1). The seed area ranged from 95.42 ± 4.05 to 218.81 ± 3.55 mm^2^ with an average of 149.42 ± 33.63 mm^2^. High values were obtained for accessions ODS-15-044, ODS-15-115, TOT6439, ODS-15-020, ODS-15-100, ODS-15-013, ODS-15-021 and BAR1807B. A lower value was obtained for accession TOT3527. The seed perimeter varied between 3.87 ± 0.05 and 6.19 ± 0.59 mm with an average of 4.90 mm. Higher values were obtained for ODS-15-020, TOT8887, ODS-15-044, ODS-15-115, ELG19/07A, BAR 1807B, ODS-15-013, ODS-15-100, ODS-15-121, while a lower value was obtained for accession TOT3527. The seed width ranged between 0.93 ± 0.05 and 1.74 ± 0.02 mm with an average of 1.29 ± 0.17 mm. Higher values were obtained in accessions ODS-15-044, ODS-15-115, TOT6439, ODS-15-013, ODS-15-020, ODS-15-100 and a lower value in the accession TOT3527. The seed length ranged from 1.19 ± 0.05 to 1.69 ± 0.01 mm with an average of 1.43 ± 0.15 mm. The accessions BAR1807B, ODS-15-044, ODS-15-020, ODS-15-115, ODS-15-121, TOT6439, TOT8887, ODS-15-100, ODS-15-15-019 showed higher values while a lower value was obtained for the accession TOT7200SC. The 10-seed weight of spider plant accessions ranged from 6.33 ± 0.58 to 17.10 ± 0.85 mg with an average of 10.44 ± 3.13 mg. The accessions with higher seed weight were ODS-15-121, ODS-15-111, TOT6439 and KSI2407A. Accession TOT7198 showed a lower seed weight. A significant difference with geographical origin was also observed for all the morphological traits (Fig. 4). In general, Asian accessions showed smaller seeds while West Africa’s accessions showed bigger seeds values.

### Mineral composition of *Gynandropsis gynandra* seeds

The screening of *G. gynandra* seeds by the Energy Dispersive Spectroscopy Solution showed a total of eight mineral elements, including carbon (C), oxygen (O), magnesium (Mg), aluminium (Al), phosphorus (P), sulphur (S), potassium (K) and calcium (Ca). Highly significant (*p* < 0.001) differences were observed among accessions with respect to all mineral elements, except for aluminium (Al) (Additional file 1). The coefficient of variation was relatively high (> 20%) for magnesium, phosphorus, sulphur, potassium and calcium. The carbon content in the seeds ranged from 55.92 ± 0.42 g / 100 g to 62.82 ± 2.04 g / 100 g with an average of 58.50 ± 1.64 g / 100 g while oxygen content ranged between 33.39 ± 1.69 g / 100 g and 40.11 ± 0.71 g / 100 g with an average of 38.04 ± 1.48 g /100 g (Additional file 1). The accessions TOT8887, BAR1807B, TOT6439 and TOT8887 showed higher carbon content while accessions ODS-15-013 and ODS-15-061 showed higher value for oxygen. Moreover, lower value for carbon was observed in the accession ODS- 15- 053 while accession TOT8887 showed the lowest oxygen value. The magnesium content of spider plant seeds ranged from 0.11 ± 0.70 g / 100 g to 0.56 ± 0.01 g /100 g with an average value of 0.30 ± 0.10 g / 100 g while the aluminium content ranged from 0.01 ± 0.02 g / 100 g to 0.70 ± 0.71 g / 100 g with an average of 0.20 ± 0.18 g /100 g. A higher value for magnesium was recorded for the accession ODS-15-053 while aluminium was higher for accession TOT8887. Phosphorus and sulphur content in the accessions ranged from 0.15 ± 0.07 g / 100 g to 0.59 ± 0.13 g / 100 g and from 0.33 ± 0.10 g / 100 g to 1.17 ± 01.4 g / 100 g with an average of 0.30 ± 0.11 g /100 g and 0.69 ± 0.17 g /100 g respectively. Higher phosphorus and sulphur values were both recorded for the accession ODS-15-053. Potassium content in Spider plant seed ranged from 0.08 ± 0.05 g / 100 g to 2.58 ± 1.25 g /100 g while the calcium content ranged from 0.42 ± 0.20 to 1.79 ± 0.24 g / 100 g with an average of 1.05 ± 0.67 g / 100 g for potassium and 0.92 ± 0.35 g / 100 g for calcium. Accession ODS-15-019, showed higher potassium content, while ELG 1907A showed higher calcium content.

**Fig. 5:**
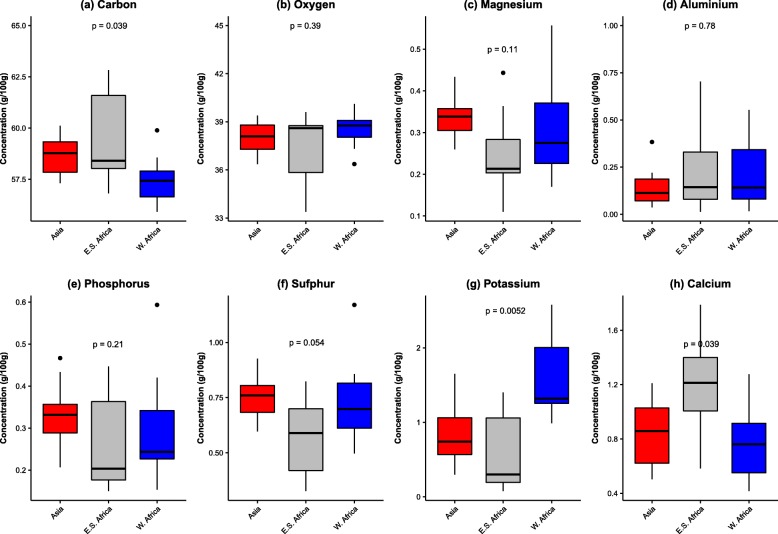
Seed mineral content variation among Asian (*n = 10*), East–Southern African (*n* = 9) and West African (*n* = 10) accessions of *Gynandropsis gynandra*

Geographical origin affected the variation of mineral elements such as carbon, potassium and calcium (Fig. 5). For instance, accessions from West Africa contained a higher amount of potassium (*p* = 0.0052) compared with other regions. Accessions from Asia were richer in carbon and accessions from East-southern Africa exhibited higher calcium content.

### Variation in seed germination

**Fig. 6:**
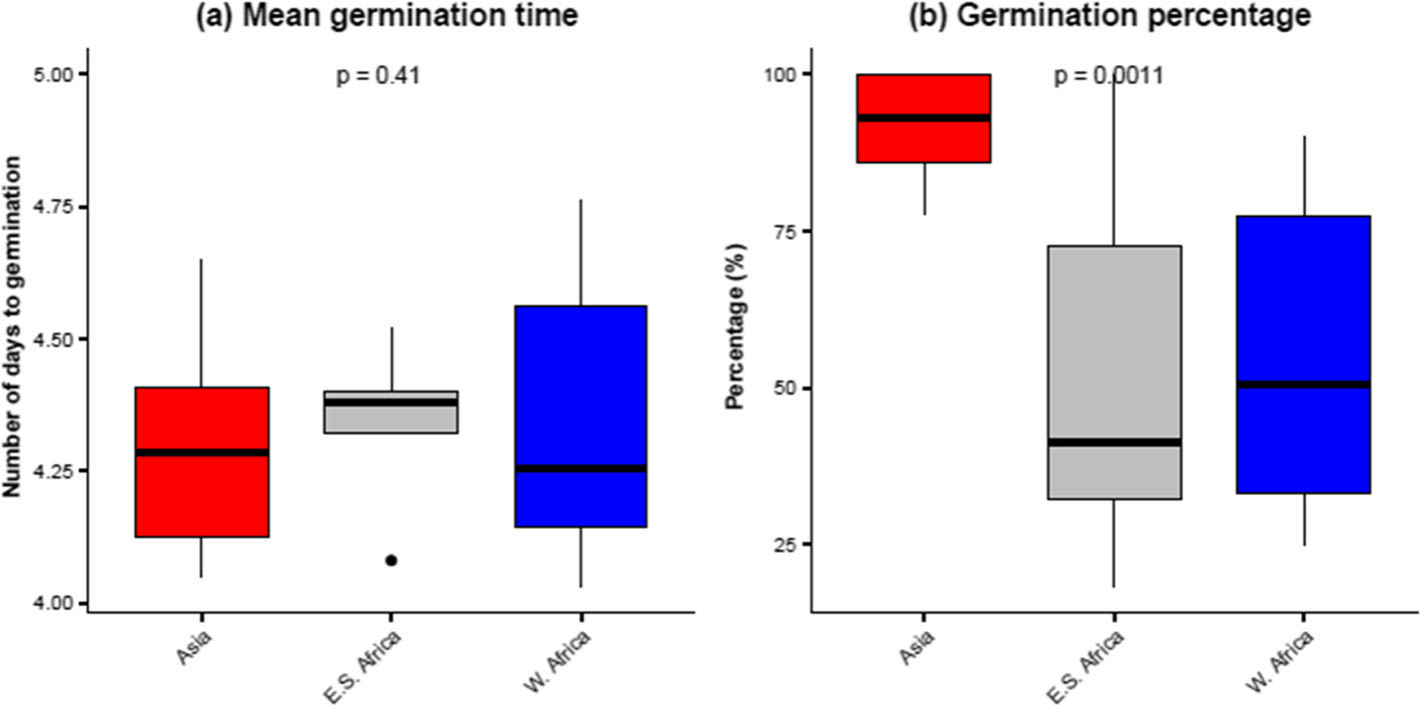
Variation in (**a**) mean germination time and (**b**) germination percentage among Asian (*n = 10*), East–Southern African (*n* = 9) and West African (*n* = 10) accessions of *Gynandropsis gynandra*

Prior seed viability test with 2,3,5-triphenyltetrazolium chloride on selected accessions from various origins revealed high viability percentage in *Gynandropsis gynandra* accessions. Viability percentages were 80% in TOT8887, 82.5% in ELG1907A, 92.5% in ODS-15-020, and 95% in TOT5799SC. Generalized linear model (GLM) analysis of the mean germination time and the percentage germination in spider plant showed highly significant differences (*p* < 0.001) among accessions (Supplementary file 1). The mean germination times ranged from four to five days with most accessions geminated within four days. Seed germination percentages after seven days varied from 24.24 ± 10.50 to 100%. Five Asian accessions obtained 100% germination including TOT1048, TOT3527, TOT6439, TOT7198 and TOT7505. Lower germination percentages were observed from the East African accession KF07 (24.24 ± 10.50%). The coefficient of variation was high for the germination percentage (cv = 43.57%), which was indicative of implied diversity among the accessions used in this study. Moreover, the percentage of germination varied with geographical origin (Fig. 6). Asian accessions germinated better than accessions from other regions. The lowest germination percentage observed in Asian genotypes was 80% in this study.

### Correlation among seed traits, germination parameters and mineral composition

The Pearson correlation analysis among germination percentage, mean germination time, morphological traits and mineral element contents of spider plant revealed highly significant, moderate, and negative correlations between seed area (r = − 0.52), seed perimeter (r = − 0.58), seed length (r = − 0.53) and 10-seed weight (r = − 0.58), seed width (r = − 0.45) with magnesium content (Table 2). Moderate and negative correlations were observed between 10-seed weight (r = − 0.53), mean germination time (r = − 0.49) and sulphur content (Table 2). Likewise, significant moderate and negative correlations were also detected between 10-seed weight (r = − 0.52), seed area (r = − 0.47), seed width (r = − 0.45), mean germination time (r = − 0.44) and phosphorus content. A significant, low and negative correlation was observed between phosphorus content and seed perimeter (r = − 0.37). In contrast, a significant moderate and positive correlation was observed between calcium content and 10-seed weight (r = 0.47).

**Table 2:** Pearson correlation analysis between size, germination parameters and mineral content of *Gynandropsis gynandra* seeds

Variables	Mg	P	S	Ca	MGT	%germination
Seed area	−0.52***	− 0.47*	− 0.26^ns^	0.33^ns^	− 0.16^ns^	−0.20^ns^
Seed perimeter	−0.58***	−0.37*	− 0.17^ns^	0.32^ns^	− 0.32^ns^	−0.19^ns^
Seed width	−0.47*	−0.45*	− 0.20^ns^	0.3^ns^	− 0.17^ns^	−0.26 ^ns^
Seed length	−0.53***	−0.43*	− 0.24^ns^	0.34^ns^	− 0.18^ns^	−0.14 ^ns^
10-Seed weight	−0.58***	−0.52***	− 0.53***	0.47*	0.13 ^ns^	−0.23 ^ns^
MGT	−**0.29**^**ns**^	−0.44*	− 0.49*	0.11^ns^	1.00	0.38 ^ns^
% germination	−**0.19**^**ns**^	−0.06^ns^	0.00^ns^	−0.10^ns^	0.38^ns^	1.00

*** *p* < 0.001, *ns* non-significant, *Mg* Magnesium, *P* Phosphorus, *S* Sulphur, *Ca* Calcium, *MGT* Mean germination time, %germination = germination percentage

### Differentiation among *G. gynandra* accessions based on principal components and hierarchical cluster analyses

**Fig. 7:**
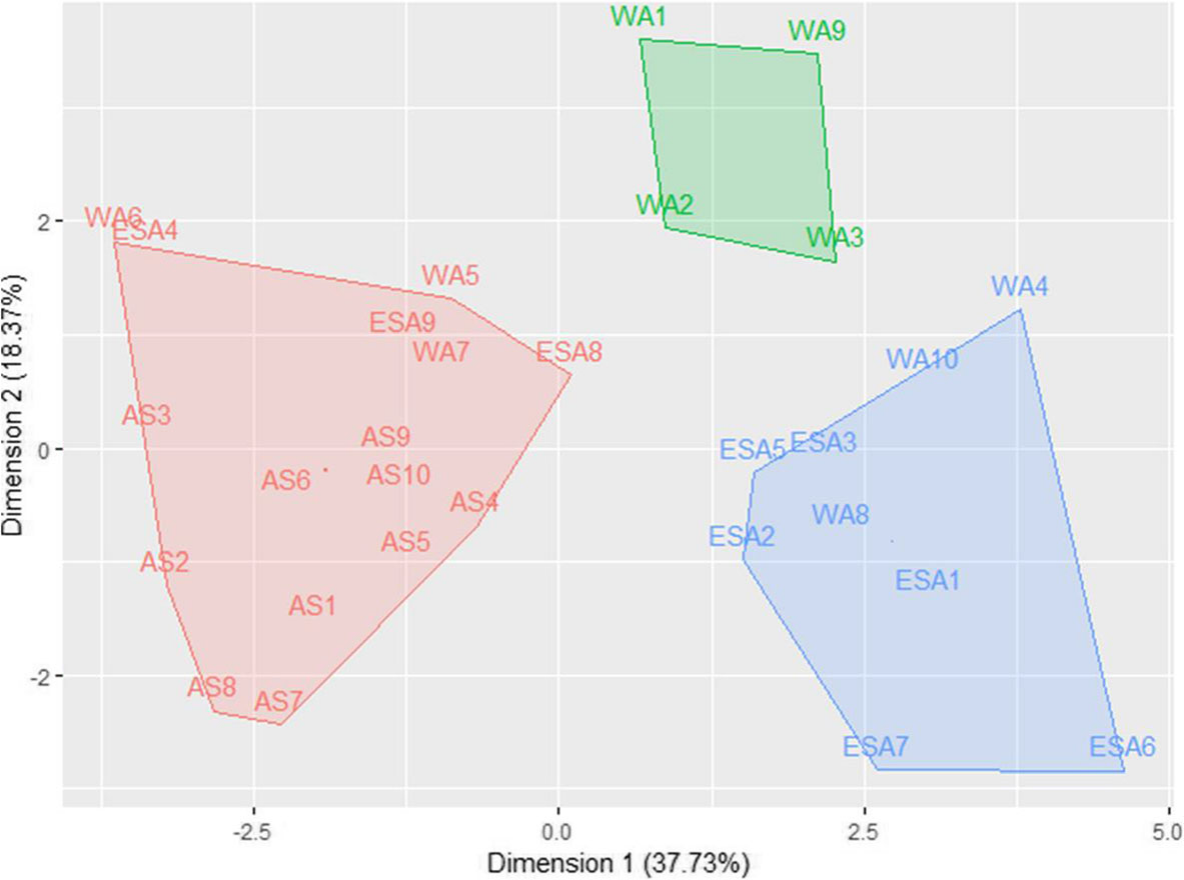
Clustering of 29 *Gynandropsis gynandra* accessions based on seed morphology, mineral content and germination: Cluster 1 (red); Cluster 2 (blue) and Cluster 3 (green)

The principal components analysis using morphological traits, germination percentage, mean germination time and mineral content of spider plant seeds revealed that the three first components explained 69.25% of the total variation (Fig. 7). The first principal component axis (Dimension 1) explained 37.66% of variation and was highly correlated with magnesium, phosphorus, calcium, seed area, seed perimeter, seed width, seed length and seed weight. The second principal component axis (Dimension 2) explained 18.73% of the variation and was highly correlated with carbon, oxygen, and potassium content, mean germination time and germination percentage. The third principal component axis (Dimension 3) explained 13.14% and was highly correlated with aluminium and sulphur content (Table 3).

**Table 3:** Correlation between variables and the three first principal components based on seed mineral content, seed size and weight, and seed germination parameters

Variables	Unit	Principal Components		
		1	2	3
Carbon	g/100 g	0.27	**−0.80**	0.43
Oxygen	g/100 g	−0.28	**0.67**	−0.57
Magnesium	g/100 g	**−0.78**	0.36	0.21
Aluminium	g/100 g	0.24	−0.19	**0.55**
Phosphorus	g/100 g	**−0.66**	0.23	0.45
Sulphur	g/100 g	−0.53	0.26	**0.60**
Potassium	g/100 g	−0.05	**0.50**	−0.11
Calcium	g/100 g	**0.57**	−0.23	−0.14
Seed area	mm^2^	**0.90**	0.35	0.08
Seed Perimeter	mm	**0.87**	0.18	0.38
Seed width	mm	**0.85**	0.38	0.09
Seed length	mm	**0.88**	0.23	−0.18
10-Seed weight	mg	**0.85**	0.23	−0.18
Mean Germination Time	day	−0.11	**0.55**	0.50
Percentage of germination	%	−0.12	−**0.59**	− 0.18-

Significant values are indicated in bold.

The Principal Components Analysis (PCA) grouped the 29 spider plants accessions into three major clusters (Fig. 7). Cluster 1 were highly separated from Cluster 2 and 3 (overall ANOSIM R = 0.5, Table 4) and discriminated by morphological traits (seed area, seed length, seed perimeter, 10-seed weight and seed width), mean germination time and mineral content (magnesium, phosphorus, sulphur, calcium, carbon, oxygen, and potassium).

**Table 4:** Results of the pairwise analysis of similarity among clusters

Overall test			
Overall R^a^ = 0.5***			
Clusters	1	2	3
1	0		
2	0.74***	0	
3	0.5***	0.47^ns^	0

^a^Statistical test to measure the degree of separation of the clusters; ***p < 0.001, *ns* non-significant

Based on the PCA results, Cluster 1 was composed of 55.17% of spider plant accessions (Table 5). Seeds of those accessions were characterized by higher magnesium (0.36 ± 0.07 g/100 g), phosphorus (0.35 ± 0.10 g/100 g), and sulphur (0.75 ± 0.15 g/100 g) content. In addition, accessions of this cluster presented the lowest values for morphological traits including seed area (125.35 ± 16.88), seed perimeter (4.44 ± 0.33), seed width (1.18 ± 0.11), seed length (1.33 ± 0.08) and seed weight. Cluster 2 consisted of 17.24% of spider plant accessions. The seeds had higher oxygen (38.58 ± 1.16) and potassium (1.72 ± 0.61) content. In contrast to Cluster 1, the seeds of accessions in Cluster 2 had highest values for all morphological traits including seed area (193.57 ± 23.9), seed perimeter (5.61 ± 0.44), seed width (1.50 ± 0.15), seed length (1.61 ± 0.09) and 10-seed weight (13.33 ± 1.13). In contrast to Clusters 1 and 2, Cluster 3 grouped 27.58% of accessions with higher carbon (59.95 ± 1.93) and calcium (1.30 ± 0.33) content. Cluster 3 was also composed of accessions with higher seed perimeter (5.36 ± 0.37) and seed width (1.38 ± 0.07). No significant differences were observed among clusters regarding the seed germination percentage, although accessions in Cluster 1 exhibited relatively 16% higher values (73.78 ± 27.93).

**Table 5:** Description of clusters of *Gynandropsis gynandra* accessions based on the PCA results

Variables	Cluster 1	Cluster 2	Cluster 3	F value
	*N* = 16	*N* = 5	*N* = 8	
Carbon (g/100g)	58.26 ± 1.07^b^	56.95 ± 0.76^b^	**59.95 ± 1.93**^**a**^	0.001
Oxygen (g/100g)	**38.30 ± 1.02**^**a**^	**38.99 ± 0.76**^**a**^	36.91 ± 2.01^b^	0.021
Magnesium (g/100g)	**0.36 ± 0.07**^**a**^	0.28 ± 0.08^b^	0.20 ± 0.05^c^	*p* < 0.001
Aluminium (g/100g)	0.16 ± 0.15	0.19 ± 0.16	0.29 ± 0.23	0.211
Phosphorus (g/100g)	**0.35 ± 0.10**^**a**^	0.2 ± 0.1^ab^	0.22 ± 0.08^b^	0.008
Sulphur (g/100g)	**0.75 ± 0.15**^**a**^	**0.74 ± 0.09**^**a**^	0.53 ± 0.17^b^	0.006
Potassium (g/100g)	1.06 ± 0.47^b^	**1.78 ± 0.67**^**a**^	0.59 ± 0.68^b^	0.004
Calcium (g/100g)	0.76 ± 0.24^b^	0.82 ± 0.27^b^	**1.30 ± 0.33**^**a**^	*p* < 0.001
Seed area (mm^2^)	125.35 ± 16.88^c^	**193.57 ± 23.9**^**a**^	169.97 ± 19.7^b^	*p* < 0.001
Seed perimeter (mm)	4.44 ± 0.33^b^	**5.61 ± 0.44**^**a**^	5.36 ± 0.37^a^	*p* < 0.001
Seed width (mm)	1.18 ± 0.11^b^	**1.50 ± 0.15**^**a**^	1.38 ± 0.07^a^	*p* < 0.001
Seed length (mm)	1.33 ± 0.08^c^	**1.61 ± 0.09**^**a**^	1.54 ± 0.12^b^	*p* < 0.001
10-seed weight (mg)	8.16 ± 1.78^b^	**13.33 ± 1.13**^**a**^	13.17 ± 2.3^a^	*p* < 0.001
Mean germination time (day)	4.33 ± 0.2	4.43 ± 0.27	4.26 ± 0.14	0.316
Percentage of germination (%)	73.78 ± 27.93	53.68 ± 29.28	57.49 ± 28.27	0.254

Values in bold indicate the cluster in which each variable was high. *N* = number of accessions. Letters a, b, and c indicate post-hoc comparisons among clusters.

**Fig. 8:**
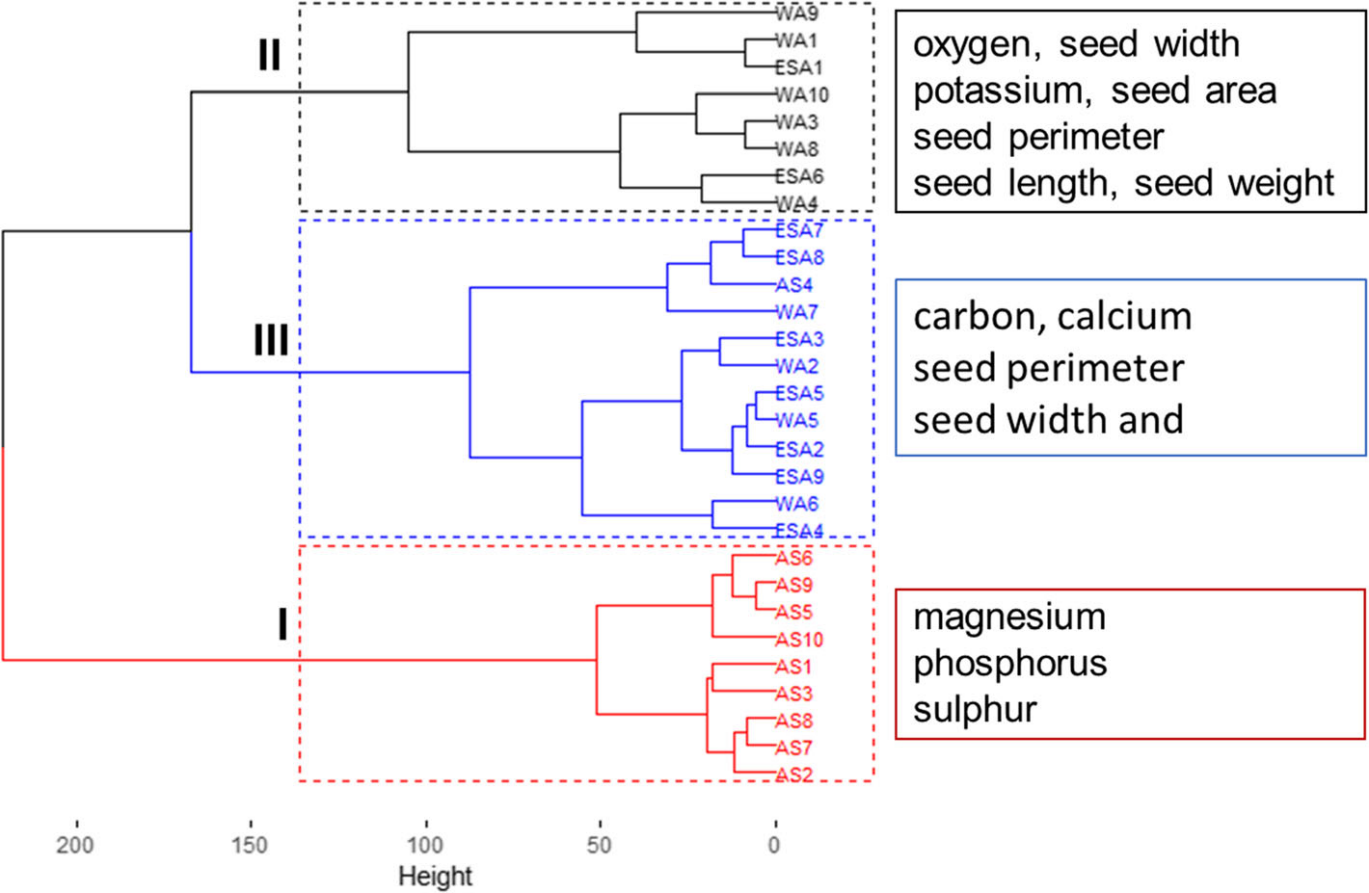
Dendrogram of 29 accessions of *Gynandropsis gynandra* from WA: West Africa, EA: East Africa, SA: South Africa and AS: Asia based on seed morphology, germination and mineral composition with the discriminant traits of each cluster with each cluster higher discriminant traits

The results of the Hierarchical Clusters Analysis (HCA) were illustrated with Fig. 8, which also gathered accessions into three clusters (A, B and C) with the composition of each cluster slightly different from the PCA clusters. The analysis of the dendrogram revealed that accessions were partially grouped based on their geographical origin and germination potential. Cluster A was composed of Asian accessions only with high germination percentage (93.75 ± 6.95), while Cluster B consisted mainly of West African accessions with higher seed size but also included one accession from East African and one from South Africa. Cluster C was composed of East African accessions (6), west African accessions (4), south African accession (1) and Asian accession (1). Analysis of variance based on the dendrogram classification did not show any differences between the three clusters with regards to mineral elements (Supplementary file 2).

## Discussion

The morphological traits of the seeds are relevant for taxonomic identification of plant species and the assessment of the existing genetic diversity within crop species [26, 27]. This study investigated seed morphology, mineral composition and germination potential of 29 *Gynandropsis gynandra* accessions from West Africa, East-southern Africa and Asia.

The study established the presence of eight (8) mineral elements, including carbon (C), oxygen (O), magnesium (Mg), aluminium (Al), phosphorus (P), sulphur (S), potassium (K) and calcium (Ca) in the seeds of *G. gynandra* accessions. One hundred gram (100 g) of dry mature seeds of *G. gynandra* contain on average 58 g of carbon, 38.04 g of oxygen, 0.30 g of magnesium, 0.20 g of aluminium, 0.30 g of phosphorus, 0.69 g of sulphur, 1.05 g of potassium and 0.92 g of calcium. These amounts are generally higher compared to those in *Amaranth* grain which contains calcium (0.0783 to 1.0046 g), iron (0.00361 to 0.02251 g), magnesium (0.04431 to 0.09738 g), potassium (0.2678 to 0.4736 g) and zinc (0.00053 to 0.00123 g) [28]. We observed, however, that the germination ability of the species was not correlated to any of the mineral elements identified in the seed. The function of those mineral elements can be further investigated in relation with the pools of unbound metabolites and solutes produced by the degradation of storage reserves during the embryo growth and the radicle protrusion [29]. More so, further investigation could focus on the analysis of the oil, amino acid, starch or oligosaccharides profile of the seeds to complement the mineral elements content. For instance, the Kew Seed Information Database (https://data.kew.org/sid) mentioned values of 25.5% oil content and 18.5% protein content for the species. However, we assume that using different accessions would give different results with the differences observed across regions.

Morphological traits including seed area, seed perimeter, seed length, seed width and 10-seed weight were significantly different among accessions. The results also showed significant differences among the accessions with respect to germination percentage and the mean germination time. In addition, the coefficient of variation was high for the germination percentage, thus suggesting the existence of phenotypic diversity among accessions. Initial seed viability was high for the randomly selected four accessions used as control. However, the Asian accessions showed higher viability percentage (95%) than the accessions from West Africa (92.5%) and East Africa (< 83%). This confirmed the results of the germination test which followed the same trend. However, we observed a huge difference between the viability percentage of seeds from East Africa and their germination percentages (up to 70% difference). We concluded that the difference observed in germination percentages of *Gynandropsis gynandra* accessions from Asia, East Africa and West African was due to dormancy. Various dormancy breaking methods can be further investigated to confirm our findings.

Baskin, Baskin [30] explained that in general the number of plant species that may acquire dormancy tend to increase with geographical distance from the equator and correlates with the occurrence of seasons. Dormancy variation can also be found within plant species. In *Arabidopsis thaliana*, it has been observed that accessions from southern Europe and parts of Asia show a tendency for higher seed dormancy levels compared with accessions from northern Europe [27]. In our case, spider plant accessions from Asia showed lower dormancy levels compared with accessions from Africa. This result complemented the explanation provided by Baskin, Baskin [30].

The identification of *Gynandropsis gynandra* specific seed dormancy regulators can significantly enhance the germination capacity of the species and its adoption. Seed germination and dormancy regulation in plants is controlled by several factors including proteins, plant hormones (abscissic and gibberellic acid), chromatin related factors (e.g. methylation, acetylation, histone), related genes (maturing genes, hormonal and epigenetics regulating genes) [31, 32]. Future research could investigate hormonal signalling during seed germination and seed biology. Genetic and molecular dormancy investigation in the species could also be performed as realized in *Arabidiospsis thalania* [33, 34] to study the gene expression profiles in different accessions of *Gynandropsis gynandra* based on the observed variation about dormancy.

Genetic variation has been studied in many species by crossing genotypes with different dormancy levels, followed by the analysis of their progeny and parallel selection for seed dormancy [33, 35, 36]. The analysis of association patterns among morphological seed traits, mineral elements and germination parameters revealed significant negative correlations between seed size (i.e. area, length, perimeter, width), 10-seed weight and mean germination time with phosphorus content. Seeds with high phosphorus content showed a reduced mean germination time, thus suggesting that phosphorus plays an important role during germination. Based on the results in this study, the hypothesis that larger seeds germinate faster/ better than smaller seeds is rejected because no correlation was observed between seed size, germination percentage and mean germination time. However, even though no correlations between seed size and germination percentage were observed, the Asian accessions which had the smallest seed size germinated better than those from East and West Africa. Similar results were reported in pea (*Pisum sativum* L.) where seeds with low seed weight showed higher germination percentage than those with higher seed weight [37]. In this study, *Gynandropsis gynandra* accessions from Asia had smaller seed size and weight and also showed the highest germination percentage. These findings imply that seed dormancy and bottlenecks in seed germination of *Gynandropsis gynandra* could be improved through crosses among the best accessions based on seed size and germination percentage.

Phenotypic diversity assessment is important to depict the extent of genetic diversity within crop species for the development and deployment of improved varieties with farmers’ desired traits [38, 39]. Our results showed three clusters of accessions highly discriminated by seed parameters including seed area, seed length, seed perimeter, seed width, 10-seed weight, mean germination time, magnesium, phosphorus, sulphur, calcium, carbon, oxygen, and potassium. The clustering was also partially explained by the geographical origin of accessions as revealed by Sogbohossou et al. [20] that focused on plant morphology and leaf nutrients content. For example, the authors found that Asian accessions were characterized by short plants with broad leaves [20] whereas in our case Asian accessions are characterized by small seed size, high phosphorus content and better germination percentage. Further studies could investigate the correlation between morphological characteristics of the seed and the leaf yield of *Gynandropsis gynandra* to develop a selection index for higher yielding leaf accessions based on the morphological traits of the seeds. Moreover, based on our results, accessions with extreme values can be used to develop mapping populations for seed related traits including fatty acid, protein, and gibberellic acid content. Seed traits could be incorporated into breeding programmes for an effective improvement of *Gynandropsis gynandra* as reported by Mohammed et al. [40] on Bambara groundnut (*Vigna subterranea* [L.] Verdc.). Finally, the phenotypic diversity observed among *G. gynandra* accessions for seed metric parameters, seed germination and mineral content can be used to improve the germination capacity of the species as well as the yield of the species.

## Conclusion

This study has generated useful information about the internal and external seed morphology as well as mineral composition of *Gynandropsis gynandra* seeds. Seed mineral composition differed significantly among different accessions except for aluminium. The relatively high-level of dissimilarity observed among clusters, and especially among accessions from different geographical areas provides a basis for the identification of desirable parents. This can be used to create segregating populations and better possibilities for genetic improvement of the crop. The diversity observed among spider plant accessions using seed attributes was also an indicator that a systematic selection of spider plant accessions into homogenous groups of seeds could be done for an effective breeding to boost seed quality, crop productivity and nutritional security.

## Methods

### Plant materials

Twenty-nine (29) accessions from three different geographic regions, namely, West Africa, East-Southern Africa and Asia were used in the study (Table 6). Seeds from these accessions were harvested in 2017 from an experimental site at the Faculty of the Agronomic Sciences (FSA) of the University of Abomey Calavi in Benin and stored for 15 months before the start of this experiment. The species was grown during the rainy season from April to July 2017 during which the temperature varied between 25.6 °C and 28 °C, the average rainfall varied between 137 and 356 mm monthly and the relative humidity oscillated between 72.5 and 82.5% (https://www.weatheratlas.com/en/benin/cotonou-climate). We harvested three plants per accession.

**Table 6:** Origin and institutional provenance of accessions used for seed morphology traits and mineral composition in *Gynandropsis gynandra*. KENRIK: Kenya Resource Center for Indigenous Knowledge; WVC: World Vegetable Center; GBioS: Laboratory of Genetics, Horticulture and Seed Science

Code	Accession	Institution	Origin	Region
ESA1	BAR 1807B	KENRIK	Kenya	East-Southern Africa
ESA2	ELG 19/07A	KENRIK	Kenya	East-Southern Africa
ESA3	HBY/2307b	KENRIK	Kenya	East-Southern Africa
ESA4	KF-07	KENRIK	Kenya	East-Southern Africa
ESA5	KSI 2407A	KENRIK	Kenya	East-Southern Africa
ESA6	TOT6439	WVC	Zambia	East-Southern Africa
ESA7	TOT8887	WVC	Uganda	East-Southern Africa
ESA8	TOT8926	WVC	Kenya	East-Southern Africa
ESA9	TOT8931	WVC	South Africa	East-Southern Africa
WA1	ODS-15-013	GBioS	Benin	West Africa
WA2	ODS-15-019	GBioS	Benin	West Africa
WA3	ODS-15-020	GBioS	Benin	West Africa
WA4	ODS-15-044	GBioS	Benin	West Africa
WA5	ODS-15-045	GBioS	Togo	West Africa
WA6	ODS-15-053	GBioS	Togo	West Africa
WA7	ODS-15-061	GBioS	Togo	west Africa
WA8	ODS-15-100	GBioS	Togo	West Africa
WA9	ODS-15-115	GBioS	Ghana	West Africa
WA10	ODS-15-121	GBioS	Ghana	West Africa
AS1	TOT1048	WVC	Thailand	Asia
AS2	TOT3527	WVC	Lao People’s Democratic Republic	Asia
AS3	TOT3536	WVC	Lao People’s Democratic Republic	Asia
AS4	TOT4976	WVC	Thailand	Asia
AS5	TOT5799	WVC	Thailand	Asia
AS6	TOT7196	WVC	Malaysia	Asia
AS7	TOT7198	WVC	Malaysia	Asia
AS8	TOT7200SC	WVC	Malaysia	Asia
AS9	TOT7486	WVC	Lao People’s Democratic Republic	Asia
AS10	TOT7505	WVC	Lao People’s Democratic Republic	Asia

### Seed morphology traits and mineral composition

Morphological and mineral element studies were performed at the Microscopic Microanalysis Unit (MMU) and the Phytopathology Laboratory of the University of KwaZulu-Natal, College of Agriculture, Engineering and Science. Scanning Electron Microscope (SEM) images were obtained with Zeiss Microscopy (EVO/LS15) of the Microscopic Microanalysis Unit (MMU). Seed samples were mounted on copper stubs on a double sided adhesive carbon tape and placed at different positions to facilitate observation. SEM images of three seeds per accessions were used to determine their dimension (images analysis). Fractured seeds (longitudinally and vertically) were used to study the seed anatomy. To complement seed description, the mineral elements analysis of seeds was done using the Integrated Energy Dispersive Spectroscopy Solution (EDS) of the Zeiss Microscopy (EVO/LS15).

To further describe the internal structure of the seeds, observations were made using a Binocular zoom stereo microscope (Carl Zeiss Stemi SV6) equipped with an Electronic Light source (Schott KL 1500) and with a digital microscope camera.

The imaging analysis was performed on individual seeds using the digital image analysis software (AnalysisSIS) at the Microscopic Microanalysis Unit. The system was calibrated to millimeters under 100x magnification and 200 μm scale before the measurements. The variables measured on imported SEM images included: 1) Seed width (SW): the distance between two points stretching from the base of the embryo axis to the tip of the endosperm of the seed (Fig. 1 b in green); 2) Seed length (SL): the length of the line drawn across the widest section of the seed (Fig. 1 b in red); 3) Seed area (A) and perimeter (Pe) were directly obtained after drawing a circle around the seed touching all edges.

The 10-seed weight was recorded using the average weight of samples of 10 seeds randomly chosen and weighed using a precision balance Ohaus® Pioneer™ Plus analytical balance Model PA114C, AC/DC input 230 V AC, universal plug set measuring up to four decimals. The 10-seed weight was duplicated four times for each accession.

The germination capacity of the seeds was investigated using 50 seeds in a petri dish and placing them in an incubator at a temperature of 30 °C under dark conditions. Each treatment (accession) was replicated 4 times. The number of newly germinating seeds was counted each day for seven days and used to calculate the mean germination time (eq. 1) and the percentage germination.

$$ MGT=\left(\Sigma \mathrm{nidi}\right)/\Sigma\ \mathrm{N}\Big)\ (1) $$

where *ni* = the number of germinated seeds at day *i*, *di* = incubation period in days, and *N* = number of germinated seeds in test.

**Fig. 9:**
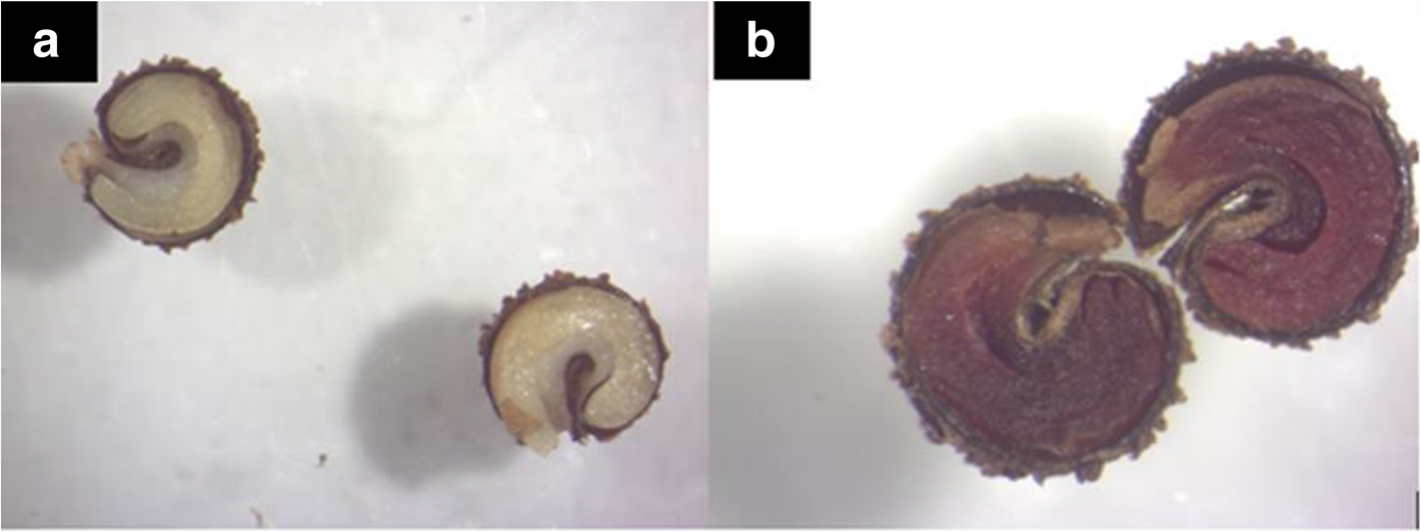
Light microscopy images of spider plant seed cross section showing how its stain with a tetrazolium solution; **a**: before tetrazolium test **b**: Viable seed after tetrazolium test (scale 200 μm)

The tetrazolium viability test, described by the International Seed Testing Association (ISTA) book [41], was done to ensure that the seeds were viable before conducting germination tests. However, due to the limited number of available seeds, a sample of four accessions from Asia, East and West Africa (including TOT 8887, TOT5499, ODS-15-020 and ELG 19078) were used with four replicates of ten seeds per accessions. Seeds were imbibed in distilled water for 24 h at 20 °C and cut into two parts before 1.0% of 2,3,5 triphenyl tetrazolium chloride solution (TZ) were added. After 24 h of incubation at 30 °C the number of viable and non-viable seeds were identified by the staining of the embryo and counted under a light microscope at the Plant Pathology laboratory of the University of KwaZulu-Natal (Fig. 9).

### Data analysis

Descriptive analysis was conducted to show trends in morphological traits, germination and mineral elements. Analysis of variance (ANOVA) was carried out on all quantitative variables to describe the variation among accessions except for the mean germination time and percentage of germination that were compared using a generalised linear model with quasi-poisson error structure. The Kruskal-Wallis tests was used as appropriate to compare the mean germination time and the percentage of germination of accessions from different regions. Means were separated using Least Significant Difference (LSD 5%) for seed morphological trait and mineral element contents. Pearson correlation analysis was done to show the linear correlation among morphological traits, mineral content and germination parameters. In addition, principal components analysis (PCA) and hierarchical cluster analysis (HCA) were performed to group the 29 accessions into different clusters using seed morphological traits, germination parameters and mineral element contents with the R package “FactoMiner” [42]. A dendrogram was generated using the function “hclust” of the R package “vegan” [43] to analyse the relationship among the accessions. All the data were analyzed using R software version 3.5.1 [44].

## Supplementary information

**Additional file 1. **The file presents the list of accessions included in the study, their geographic origins and codification name. This file included also the mean and standard deviation for all morphological traits, mineral element contents and germination parameters of all *Gynandropsis gynandra* accessions used in the study.

**Additional file 2.** The file presents the description of each cluster based on the dendrogram results

## Data Availability

All data generated or analysed in this study are included within the article or supplementary materials. The raw datasets and R scripts generated during the current study are available from the corresponding author on reasonable request. The seeds used in this study can be requested from the Laboratory of Genetics, Horticulture and Seed Science, Faculty of Agronomic Sciences, University of Abomey-Calavi, BP 2549, Abomey-Calavi, Republic of Benin or from the Gene bank of the Biosystematics Group, Wageningen University, Droevendaalsesteeg 1, 6708 PB Wageningen, The Netherlands using same accessions names.

## Abbreviations

Al: Aluminium
ANOVA: Analysis of variance
B: Boron
C: Carbon
Ca: Calcium
Co: Cobalt
Cu: Copper
EDS: Energy Dispersive Spectroscopy
GLM: Generalized Linear Model
HCA: Hierarchical Clusters Analysis
ISTA: International Seed Testing Association
K: Potassium
LSD: Least Significant Difference
Mg: Magnesium
MGT: Mean Germination Time
MMU: Microscopic Microanalysis Unit
Mo: Molybdenum
O: Oxygen
P: Phosphorus
PCA: Principal Components Analysis
S: Sulphur
Se: Selenium
SEM: Scanning Electron Microscopy
Zn: Zinc
