# Supplementary material for: Origin-dependence of variation in seed morphology, mineral composition and germination percentage in Gynandropsis gynandra (L.) Briq. accessions from Africa and Asia
Source: BMC Plant Biol. 2020 Apr 15;20:168. doi: 10.1186/s12870-020-02364-w (PMC7160957; doi:10.1186/s12870-020-02364-w)
Supplement: Supplementary file 2 — Additional file 2. The file presents the description of each cluster based on the dendrogram results [file 12870_2020_2364_MOESM2_ESM.docx]

| Variables | Cluster 1 | Cluster 2 | Cluster 3 | F values |
| --- | --- | --- | --- | --- |
|  | N=9 | N=8 | N=12 |  |
| Carbon | 58.65±1.07 | 58.6±2.15 | 58.33±1.74 | 0.106^ns^ |
| Oxygen | 38.01±1.05 | 37.85±1.67 | 38.19±1.73 | 0.126 ^ns^ |
| Magnesium | 0.34±0.05 | 0.25±0.09 | 0.31±0.12 | 2.208 ^ns^ |
| Aluminium | 0.15±0.11 | 0.21±0.15 | 0.23±0.23 | 0.562 ^ns^ |
| Phosphorus | 0.34±0.08 | 0.23±0.09 | 0.31±0.13 | 2.361 ^ns^ |
| Sulphur | 0.77±0.1 | 0.63±0.16 | 0.67±0.21 | 1.464 ^ns^ |
| Potassium | 0.94±0.44 | 1.23±0.8 | 1.02±0.76 | 0.3 ^ns^ |
| Calcium | 0.81±0.24 | 1.01±0.37 | 0.94±0.41 | 0.712 ^ns^ |
| Seed area | 116.4±14.24c | **194.43±15.41a** | 144.14±13.68b | 64.07*** |
| Seed perimeter | 4.34±4.34c | **5.57±0.31a** | 4.87±0.49b | 19.15*** |
| Seed width | 1.12±0.1c | **1.49±0.11a** | 1.29±0.07b | 33.77*** |
| Seed length | 1.3±007c | **1.64±0.06a** | 1.4±0.09b | 44.29*** |
| 10 seeds weigh | 7.53±0.96c | **13.57±1.96a** | 10.53±2.78b | 16.82*** |
| Mean germination time | 4.26±0.19 | 4.3±0.28 | 4.38±0.15 | 0.92 ns |
| Percentage of germination | **93.75±6.95a** | 64.72±29.51b | 45.6±20.47b | 13.99*** |

**Supplementary material 2:** Description of clusters of *Gynandropsis gynandra* accessions based of dendrogram classification.

*** p<0.001; ** p<0.01; * p<0.05; ns = non-significant. Values in bold indicate the cluster in which each variable was high. N = number of accessions
